# Genomic Bayesian Prediction Model for Count Data with Genotype × Environment Interaction 

**DOI:** 10.1534/g3.116.028118

**Published:** 2016-02-25

**Authors:** Abelardo Montesinos-López, Osval A. Montesinos-López, José Crossa, Juan Burgueño, Kent M. Eskridge, Esteban Falconi-Castillo, Xinyao He, Pawan Singh, Karen Cichy

**Affiliations:** * Departamento de Estadística, Centro de Investigación en Matemáticas (CIMAT), Guanajuato, 36240, Mexico; † International Maize and Wheat Improvement Center (CIMMYT), Apdo. Postal 6-641, 06600, México, D.F., Mexico; ‡ Department of Statistics, University of Nebraska, Lincoln, Nebraska 68583-0963; § Instituto Nacional Autónomo de Investigaciones Agropecuarias (INIAP), Panamericana Sur Km 1, Quito, Ecuador; ** Sugarbeet and Bean Research Unit, USDA-ARS, East Lansing, Michigan 48824

**Keywords:** Bayesian model, count data, genome enabled prediction, Gibbs sampler, GenPred, shared data resource, genomic selection

## Abstract

Genomic tools allow the study of the whole genome, and facilitate the study of genotype-environment combinations and their relationship with phenotype. However, most genomic prediction models developed so far are appropriate for Gaussian phenotypes. For this reason, appropriate genomic prediction models are needed for count data, since the conventional regression models used on count data with a large sample size ($n_{T}$) and a small number of parameters (*p*) cannot be used for genomic-enabled prediction where the number of parameters (*p*) is larger than the sample size ($n_{T}$). Here, we propose a Bayesian mixed-negative binomial (BMNB) genomic regression model for counts that takes into account genotype by environment (G×E) interaction. We also provide all the full conditional distributions to implement a Gibbs sampler. We evaluated the proposed model using a simulated data set, and a real wheat data set from the International Maize and Wheat Improvement Center (CIMMYT) and collaborators. Results indicate that our BMNB model provides a viable option for analyzing count data.

In most living organisms, phenotype is the result of genotype (*G*), environment (*E*) and genotype by environment interactions (G×E). Garrod (1902) observed that the effect of genes on phenotype could be modified by the environment (*E*). Similarly, Turesson (1922) demonstrated that the development of a plant is often influenced by its surroundings. He postulated the existence of a close relationship between crop plant varieties and their environment, and stressed that the presence of a particular variety in a given locality is not a chance occurrence; rather, there is a genetic component that helps the individual adapt to that area.

For these reasons, today the consensus is that *G×E* is useful for understanding genetic heterogeneity under different environmental exposures (Kraft *et al.* 2007; Van Os and Rutten 2009), and for identifying high-risk or productive subgroups in a population (Murcray *et al.* 2009); it also provides insight into the biological mechanisms of complex traits such as disease resistance and yield (Thomas 2011), and improves the ability to discover resistance genes that interact with other factors that have few marginal effects (Thomas 2011). However, finding significant *G×E* interactions is challenging. Model misspecification, inconsistent definition of environmental variables, and insufficient sample sizes are just a few of the issues that often lead to low-power and nonreproducible findings in *G×E* studies (Jiao *et al.* 2013; Winham and Biernacka 2013).

Genomics and its breeding applications are developing very quickly with the goal of predicting yet-to-be observed phenotypes, or unobserved genetic values for complex traits, and inferring the underlying genetic architecture utilizing large collections of markers (Goddard and Hayes 2009; Zhang *et al.* 2014). Also, genomics is useful when dealing with complex traits that are multigenic in nature, and have major environmental influence (Pérez-de-Castro *et al.* 2012). For these reasons, the use of whole genome prediction models continues to increase. In genomic prediction, all marker effects are fitted simultaneously on a model and simulation studies promote the use of this methodology to increase genetic progress in less time. For continuous phenotypes, models have been developed to regress phenotypes on all available markers using a linear model (Goddard and Hayes 2009; de los Campos *et al.* 2013). However, in plant breeding, the response variable in many traits is a count (*y* = 0,1,2,…), for example, number of panicles per plant, number of seeds per panicle, weed count per plot, etc. Count data are discrete, non-negative, integer-valued, and typically have right-skewed distributions (Yaacob *et al.* 2010).

Poisson and negative binomial regression are often used to deal with count data. These models have a number of advantages over an ordinary linear regression model, including a skewed, discrete distribution (0,1,2,3,…,) and the restriction of predicted values for phenotypes to non-negative numbers (Yaacob *et al.* 2010). These models differ from an ordinary linear regression model. First, they do not assume that counts follow a normal distribution. Second, rather than modeling y as a linear function of the regression coefficients, they model a function of the response mean as a linear function of the coefficients (Cameron and Trivedi 1986). Regression models for counts are usually nonlinear and have to take into consideration the specific properties of counts, including discreteness and non-negativity, and are often characterized by overdispersion (variance greater than the mean) (Zhou *et al.* 2012).

However, in the context of genomic selection, it is still common practice to apply linear regression models to these data, or to transformed data (Montesinos-López *et al.* 2015a, 2015b). This does not take into account that: (a) many distributions of count data are positively skewed, many observations in the data set have a value of 0, and the high number of 0s in the data set does not allow a skewed distribution to be transformed into a normal one (Yaacob *et al.* 2010); and (b) it is quite likely that the regression model will produce negative predicted values, which are theoretically impossible (Yaacob *et al.* 2010; Stroup 2015). When transformation is used, it is not always possible to have normally distributed data, and often transformations not only do not help, they are counterproductive. There is also mounting evidence that transformations do more harm than good for the models required by the vast majority of contemporary plant and soil science researchers (Stroup 2015). To the best of our knowledge, only the paper of Montesinos-López *et al.* (2015c) is appropriate for genomic prediction of count data in a Bayesian framework; however, it does not take into account G×E interaction.

In this paper, we extend the negative binomial (NB) regression model for counts proposed by Montesinos-López *et al.* (2015c) to take into account G×E by using a data augmentation approach. A Gibbs sampler was derived since all full conditional distributions were obtained, which allows samples to be drawn from them to estimate the required parameters. In addition, we provide all the details of the efficient derived Gibbs sampler so that it can be implemented easily by most plant and animal scientists. We illustrate our proposed methods with a simulated data set and a real data set on wheat *Fusarium* head blight. We compare our proposed models (NB and Poisson) with the Normal and Log-Normal models commonly implemented for analyzing count data. We also provide R code for implementing the proposed models.

## Materials and Methods

The data used in this study were taken from a PhD thesis (Falconi-Castillo 2014) aimed at identifying sources of resistance to *Fusarium* head blight (FHB), caused by *Fusarium graminearum*, and at identifying genomic regions and molecular markers linked to FHB resistance through association analysis.

### Experimental data

#### Phenotypic data:

A total of 297 spring wheat lines developed by the International Maize and Wheat Improvement Center (CIMMYT) was assembled and evaluated for resistance to *F. graminearum*. Phenotyping was done at CIMMYT’s El Batan experimental station in Mexico over two years (2012 and 2014), and at the Santa Catalina Experimental Station of the National Institute for Agricultural Research (INIAP), Ecuador, for one year (2014). For the application, we considered these three environments, which we named Batan 2012, Batan 2014, and Ecuador 2014. In all the experiments (environments), the genotypes were arranged in a randomized complete block design, in which each plot comprised two 1-m double rows separated by a 0.25 m space. In Ecuador 2014, the nursery was inoculated with maize seeds infected with a local *F. graminearum* isolate (SC01). The inoculum was broadcast in the field at 3 and 2 wk before anthesis, at a rate of 50 g/m^2^.

FHB severity data were collected shortly before maturity by counting symptomatic spikelets on 10 randomly selected spikes in each plot. In Mexico, plots were inoculated with a mixture of five *F. graminearum* isolates (CIMFU235, 702, 715, 720, and 770) at each line’s flowering period by spraying 30 ml of an *F. graminearum* macroconidial suspension (50,000 spores/ml) using a CO_2_-powered backpack sprayer (model T R&D Sprayers, Opelousas, LA) calibrated to 40 psi. High humidity was maintained in the field by a mist irrigation system controlled by a programmable timer that applied 10 min of spray every hour from 9:00 to 20:00. FHB severity data were collected at 25 days after inoculation by counting spikelets showing FHB symptoms on 10 spikes that had been tagged at anthesis. In this study, we used only 182 spring wheat lines because we had complete marker information only for those lines.

#### Genotypic data:

DNA samples were extracted from young leaves (2- to 3-wk-old) taken from each line, using Wizard Genomic DNA purification (Promega) following the manufacturer’s protocol. DNA samples were genotyped using an Illumina 9K SNP chip with 8632 SNPs (Cavanagh *et al.* 2013). For a given marker, the genotype for the *i*th line was coded as the number of copies of a designated marker-specific allele carried by the *i*th line (absences equal to zero, and presents equal to one). SNP markers with unexpected genotype AB (heterozygous) were recoded as either AA or BB, based on the graphical interface visualization tool of GenomeStudio (Illumina) software. SNP markers that did not show clear clustering patterns were excluded. In addition, 66 simple sequence repeat (SSR) markers were screened. After filtering the markers for 0.05 minor allele frequency (MAF), and deleting markers with more than 10% of no calls, the final set of SNPs was 1635 SNPs.

### Data and software availability

The phenotypic (FHB) and genotypic (marker) data used in this study, as well as basic R codes (R Core Team 2015), for fitting the models can be downloaded directly from the repository at http://hdl.handle.net/11529/10575.

### Statistical models

We assume that, at each environment, the *J* genotypes were grown in a randomized complete block design, and we let $y_{\text{ijkt}}$ represent the count response for the *t*th replication of the *j*th line in the *k*th block in the *i*th environment, with $i=1,...,I;j=1,2,...,J,k=1,...,K,t=1,2,...,n_{\text{ijk}}$, and we propose the following combined linear predictor for the response variable:

$$\eta_{\text{ijk}}=E_{\text{i}}+R{\left(E\right)}_{\text{ik}}+g_{\text{j}}+gE_{\text{ij}}$$ (1)

where $E_{\text{i}}$ represents environment *i*, $R{\left(E\right)}_{\text{ik}}$ represent the effect of block *k* within environment *i*, $g_{\text{j}}$ is the marker effect of genotype *j*, and $gE_{\text{ij}}$ is the interaction between markers and the environment; I=3, since we have three environments (Batan 2012, Batan 2014, and Ecuador 2014), J=182, since it is the number of lines under study, K=2, since only two blocks are available per environment, and $n_{\text{ijk}}$ represents the number of replicates of each line in each block and environment but this was the same $\left(n_{\text{ijk}}=n\right)$ for all combinations of *i*, *j* and *k n* was 10 since 10 spikes were selected at random from each plot). The number of observations in each environment *i* is $n_{i}=JKn$, while the total number of observations is $n_{\text{T}}=IJKn$. *IJ* is the product of the number of environments and number of lines. Four models were implemented using the linear predictor given in expression (1).

#### Model NB:

**Model NB** stands for model negative binomial and is defined by three distributions: $y_{\text{ijkt}}|g_{\text{j}},gE_{\text{ij}}∼\text{NB(}\mu_{\text{ijk}},r\text{)}$, with *r* being the scale parameter, ${\text{μ}}_{\text{ijk}}=\exp\left({\text{η}}_{\text{ijk}}\right),\mathrm{g=}{\left(g_{1},...,g_{\text{J}}\right)}^{T}∼N\left(0,G_{1}\sigma_{\text{g}}^{2}\right)$, and $gE_{\text{i}}={\left(gE_{\text{i}1},...,gE_{\text{iJ}}\right)}^{T}∼N\left(0,G_{2}{\text{σ}}_{\text{gE}}^{2}\right)$. Note that the NB distribution has expected value $E\left(y_{\text{ijkt}}|g_{\text{j}},gE_{\text{ij}}\right)=\mu_{\text{ijk}}$ and variance $Var\left(y_{\text{ijkt}}|g_{\text{j}},gE_{i\text{j}}\right)={\text{μ}}_{\text{ijk}}+\frac{{\text{μ}}_{i\text{jk}}^{2}}{r}$ and $Var\left(y_{\text{ijkt}}|g_{\text{j}},gE_{\text{ij}}\right)>E\left(y_{\text{ijkt}}|g_{\text{j}},gE_{\text{ij}}\right)$ for r>0. $G_{1}$ and $G_{2}$ were assumed known, with $G_{1}$ computed from marker W data(for m=1,...,q markers) as $G_{1}=\frac{WW^{T}}{q}$; this matrix is called the Genomic Relationship Matrix (GRM) (VanRaden 2008). The $G_{1}$ matrix defines the covariance between individuals based on observed similarity at the genomic level, rather than on expected similarity based on pedigree, so that more accurate predictions of merit can be achieved. While $G_{2}$ is computed as $G_{2}=I_{I}⊗G_{1}$ of order *IJ* × *IJ* and ⊗ denotes the Kronecker product, $I_{\text{I}}$ means that we assume independence between environments.

#### Model Pois:

**Figure 1:**
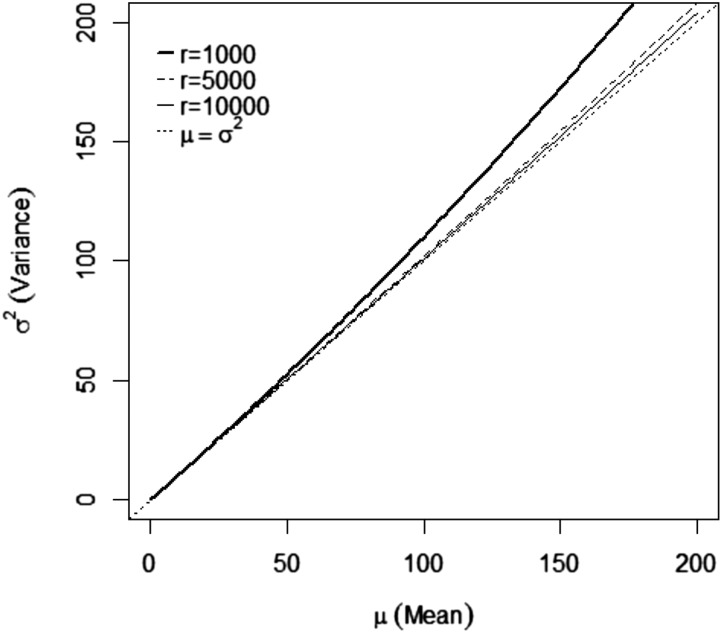
Plot of the mean count *vs.* the variance of **Model NB** as a function of the scale parameter (r). Good approximations are obtained when the mean and variance are very similar; in the plot, they should follow the diagonal that plots $\mu =\sigma^{2}.$

Model Poisson (**Model Pois**) is the same as **Model NB**, except that $y_{\text{ijkt}}|g_{\text{j}},gE_{\text{ij}}∼$ Poisson($\mu_{\text{ijk}}$). Since, according to Zhou *et al.* (2012) and Teerapabolarn and Jaioun (2014), the $\lim_{r\to \infty }\text{NB}\left({\text{μ}}_{\text{ijk}},r\right)=\text{Pois}\left({\text{μ}}_{\text{ijk}}\right),$ **Model Pois** was implemented using the same method as **Model NB**, but fixing *r* to a large value, depending on the mean count. We used r=1000, which is a good choice when the mean count is less than 50 (see Figure 1). However, when the count is between 50 and 200, we suggest using r=5000, and, when the count is larger than 200, we suggest a value of r=10,000 or larger. These suggestions are supported by Figure 1, where we plot the mean and variance of **Model NB** as a function of the scale parameter *r*, with three values of *r* (1000; 5000; 10,000). Good approximations to the **Model Pois** with the **Model NB** occur when the mean and variance are very similar. For this reason, good approximations are those that follow the diagonal in Figure 1 where $\text{μ}={\text{σ}}^{2}$. We can see that the mean count and variances are very similar for mean counts of less than 50 with r=1000; however, when the mean count is larger than 50 and less than 200, we should use r=5000, and for counts greater than 200, we suggest using a value of r=10,000 or larger. In our applications with simulated and real data, the mean count is less than 50; for this reason, we used a value of r=1000.

#### Model Normal:

**Model Normal** is similar to **Model NB,** except that $y_{\text{ijkt}}|g_{\text{j}},gE_{\text{ij}}∼\text{N(}\eta_{\text{ijk}},\sigma_{\text{e}}^{2}\text{)}$ with identity link function $\left({\text{η}}_{i\text{jk}}={\text{μ}}_{\text{ijk}}\right)$, and $\sigma_{\text{e}}^{2}$ is the scale parameter of the normal distribution and is associated with the residual in the *i* environment, *k* block, *j* line and replication *t*. The $\sigma_{\text{e}}^{2}$ parameter must be estimated since the Normal distribution, Log-normal distribution, and the Negative binomial distribution belong to the two-parameter exponential family, while the Poisson distribution belongs to the one-parameter exponential family. For this reason, only the ${\text{μ}}_{\text{ijk}}$ need to be estimated since the mean is equal to the variance. However, the scale parameter in the NB distribution is represented by *r*.

#### Model LN:

Model Log-Normal (**Model LN**) is similar to **Model NB**, except that $\log\left(y_{\text{ijkt}}+1\right)|g_{\text{j}},gE_{\text{ij}}∼\text{N(}{\text{η}}_{\text{ijk}},\sigma_{\text{e}}^{2}\text{)}$ with identity link function $\left({\text{η}}_{\text{ijk}}={\text{μ}}_{\text{ijk}}\right)$ and $\sigma_{\text{e}}^{2}$ is the scale parameter associated with the residual in the *i* environmment, *k* block, *j* line, and *t* replication.

When the number of markers (q) is larger than the number of observations $\left(n_{\text{T}}\right)$, implementing **Models NB** and **Pois** is challenging. For this reason, we propose a Bayesian method for dealing with situations when $q>n_{\text{T}}$ and our model takes into account all markers through the GRM $\left(G_{1}=\frac{{\mathrm{WW}}^{\text{T}}}{q}\right)$ described above. **Models Normal** and **LN** were implemented in the BGLR package of de los Campos *et al.* (2014). Therefore, our proposed Bayesian model for count data is a so-called Genomic Best Linear Unbiased Prediction (GBLUP) method, since it utilizes genomic relationships to predict the genetic value of an individual.

##### Bayesian mixed negative binomial regression:

Rewriting the linear predictor (1) as ${\text{η}}_{\text{ijk}}=x_{i\text{k}}^{\text{T}}\beta +\;b_{1\text{j}}+\;b_{2\text{ij}}$, with $x_{\text{ik}}^{\text{T}}=[\;x_{1},x_{2},\;x_{3},x_{11},x_{12},\;x_{21,\;}x_{22},x_{31},\;x_{32,\;}]$, where $x_{1},x_{2}\;$ and $x_{3}$ are indicator variables that take the value of 1 if the observed environment *i* is 1, 2, and 3, respectively, and 0 otherwise, $x_{\text{ik}},\;i=1,2,3$ and k=1,2,  are indicator variables that take the value of 1 if the block *k* is observed within environment *i*, and 0 otherwise. $\beta^{\text{T}}=\left[\;{\text{β}}_{1,}{\text{β}}_{2,},{\text{β}}_{3},{\text{β}}_{11,}{\text{β}}_{12,},{\text{β}}_{21},{\text{β}}_{22},\;{\text{β}}_{31,}{\text{β}}_{32}\right],$ where the first three beta coefficients belong to the effects of environment, and the last six beta coefficients correspond to the blocks effects in each of the environments (that is, **β** is a vector of beta coefficients of order p×1, with *p*=I+I×K). Therefore, $x_{\text{ik}}^{\text{T}}\beta =E_{\text{i}}+R{\left(E\right)}_{\text{ik}}\;$, $b_{1\text{j}}=g_{\text{j}}$ and $b_{2\text{ij}}=gE_{\text{ij}}$. Note that, under the **Model NB**, because ${\text{μ}}_{\text{ijk}}=E\left(y_{\text{ijkt}}|b_{1\text{j}},b_{2\text{ij}}\right)=\exp\left({\text{η}}_{\text{ijk}}\right)$, conditionally on $b_{1\text{j}}\;$ and $b_{2\text{ij}}$, the probability that the random variable $Y_{\text{ijkt}}\;$ takes the value $y_{\text{ijkt}}$ is equal to

$$\mathrm{Pr}\left(Y_{\text{ijkt}}=y_{\text{ijkt}}|b_{1\text{j}},b_{2\text{ij}}\right)=\left(\begin{array}{c} y_{\text{ijkt}}+r-1\\ y_{\text{ijkt}} \end{array}\right){\left(1-\frac{{\text{μ}}_{\text{ijk}}}{r+{\text{μ}}_{\text{ijk}}}\right)}^{r}{\left(\frac{{\text{μ}}_{\text{ijk}}}{r+{\text{μ}}_{\text{ijk}}}\right)}^{y_{\text{ijtk}}}\;\text{for}\;y_{\text{ijkt}}=0,1,2,$$

$$=\frac{\text{Γ}\left(y_{\text{ijkt}}+r\right)}{y_{\text{ijkt}}!\text{Γ}\left(r\right)}\frac{{\left[\text{exp}({\text{η}}_{\text{ijk}}^{\text{*}})\right]}^{y_{\text{ijkt}}}}{{\left[1+\text{exp}({\text{η}}_{\text{ijk}}^{\text{*}})\right]}^{y_{\text{ijkt}}+r}}\;\;\;y_{\text{ijkt}}=0,1,2,$$ (2)

We arrive at Equation (2) since we make $\frac{{\text{μ}}_{\text{ijk}}}{r+{\text{μ}}_{\text{ijk}}}=\frac{r{\text{μ}}_{\text{ijk}}}{r(r+{\text{μ}}_{\text{ijk}})}=\frac{{\text{μ}}_{\text{ijk/r}}}{{1\text{+μ}}_{\text{ijk/r}}}=\frac{\text{exp}({\text{η}}_{\text{ijk}})\text{exp}(-\log\left(r\right))}{1+\text{exp}\left({\text{η}}_{\text{ijk}}\right)\text{exp}(-\text{log}(r))}=\frac{\text{exp}({\text{η}}_{\text{ijk}}-\text{log}(r))}{1+\text{exp}({\text{η}}_{\text{ijk}}-\text{log}(r))}=\frac{\text{exp}({\text{η}}_{\text{ijk}}^{\text{*}})}{1+\text{exp}({\text{η}}_{\text{ijk}}^{\text{*}})}$, with ${\text{η}}_{\text{ijk}}^{\text{*}}=x_{\text{ik}}^{\text{T}}\beta^{*}+b_{1\text{j}}+\;b_{2\text{ij}},\;\;\beta^{*}=\left[\;{\text{β}}_{1}^{*},{\text{β}}_{2}^{*},{\text{β}}_{3}^{*},{\text{β}}_{11},{\text{β}}_{12,},{\text{β}}_{21},{\text{β}}_{22},\;{\text{β}}_{31,}{\text{β}}_{32}\right]$, and $\beta_{\text{i}}^{*}=\beta_{\text{i}}-\text{log}(r)$. Therefore, in Equation (2), we have the connection between the probability distribution of the response ($Y_{\text{ijkt}})$ induced by the assumed relation between the linear predictor ($\eta_{\text{ijk}})$ and the expected value of $Y_{\text{ijkt}}\;$ (${\text{μ}}_{\text{ijk}})$ under the **model NB**. Then we can rewrite the $\mathrm{Pr}\left(Y_{\text{ijkt}}=y_{\text{ijkt}}|b_{1j},b_{2\text{ij}}\right)\;\;\;\;$ given in Equation (2) as:

$$\frac{\Gamma \left(y_{\text{ijkt}}+r\right)}{y_{\text{ijkt}}!\Gamma \left(r\right)}2^{-y_{\text{ijkt}}-r}\text{exp}\left(\frac{y_{\text{ijkt}}-r}{2}{\text{η}}_{\text{ijk}}^{*}\right)\overset{\infty }{\underset{0}{\int }}\text{exp}\left[-\frac{{\text{ω}}_{\text{ijkt}}{\left({\text{η}}_{\text{ijk}}^{*}\right)}^{2}}{2}\right]f({\text{ω}}_{\text{ijkt}},y_{\text{ijkt}}+r,0)d{\text{ω}}_{\text{ijkt}}$$ (3)

Expression (3) was obtained using the following equality given by Polson *et al.* (2013): $\frac{{\left(e^{\text{ψ}}\right)}^{\text{a}}}{{\left(1+e^{\text{ψ}}\right)}^{\text{b}}}=2^{-\text{b}}e^{\text{κψ}}\overset{\infty }{\underset{0}{\int }}e^{-\frac{{\text{ω}}_{\text{ijkt}}\psi^{2}}{2}}f({\text{ω}}_{\text{ijkt}};b,0)d{\text{ω}}_{\text{ijkt}}$, where κ=a−b/2 and f(.,b,0) denotes the density of the Pólya-Gamma distribution (${\text{ω}}_{\text{ijkt}})$ with parameters *b* and c=0 [PG(b,c=0)] (see Definition 1 in Polson *et al.* 2013). From here, conditioning on ${\text{ω}}_{\text{ijkt}}$ $∼PG(y_{\text{ijkt}}+r,c=0)$, we have that

$$\mathrm{Pr}\left(Y_{\text{ijkt}}=y_{\text{ijkt}}|b_{1\text{j}},b_{2\text{ij}},\;{\text{ω}}_{\text{ijkt}}\right)\;\;\;\;=\frac{\text{Γ}\left(y_{\text{ijkt}}+r\right)}{y_{\text{ijkt}}!\text{Γ}\left(r\right)}2^{-y_{i\text{jkt}}-r}\text{exp}\left(\frac{y_{\text{ijkt}}-r}{2}{\text{η}}_{\text{ijk}}^{\text{*}}\right)\text{exp}\left[-{\text{ω}}_{\text{ijkt}}{\left({\text{η}}_{\text{ijk}}^{\text{*}}\right)}^{2}/2\right]$$ (4)

To be able to get the full conditional distributions, we provide the prior distributions, f(θ), for all the unknown model parameters $\theta =(\beta^{*}$, $\sigma_{\beta }^{2}$, $b_{1}$, ${\text{σ}}_{\text{b1}}^{2}$, $b_{2}$, ${\text{σ}}_{\text{b2}}^{2},$ *r*). We assume prior independence between the parameters, that is,

$$f\left(\theta \right)=f\left(\beta^{*}\right)f\left(\sigma_{\beta }^{2}\right)f\left(\;b_{1}\right)f\left(\sigma_{b1}^{2}\right)f\left(\;b_{2}\right)f\left(\sigma_{b2}^{2}\right)f\left(r\right).$$

We assign conditionally conjugate but weakly informative prior distributions to the parameters because we have no prior information. Prior specification in terms of $\beta^{*}\;$ instead of **β** is for convenience. We adopt proper priors with known hyper-parameters whose values we specify in model implementation to guarantee proper posteriors. We assume that $\beta^{*}|\sigma_{\beta }^{2}∼N_{p}\left(\beta_{0},\underset{0}{\sum }\sigma_{\beta }^{2}\right),$ $\sigma_{\beta }^{2}∼\chi^{-2}\left(\nu_{\beta },S_{\beta }\right)\;\text{where}\;\chi^{-2}\left(\nu_{\beta },S_{\beta }\right)\;$ denotes a scaled inverse chi-square distribution with shape $\nu_{\beta }\;$ and scale $S_{\beta }$ parameters, $b_{1}|\sigma_{b1}^{2}∼N_{nb1}\left(0,G_{1}\sigma_{b1}^{2}\right)$, nb1=J, $\sigma_{b1}^{2}∼\;\chi^{-2}\left(\nu_{b1},S_{b1}\right)$, $b_{2}|\sigma_{b2}^{2}∼N_{nb2}\left(0,G_{2}\sigma_{b2}^{2}\right)$, nb2=IJ, $\sigma_{b2}^{2}∼\;\chi^{-2}\left(\nu_{b2},S_{b2}\right)$ and $r∼G\left(a_{0},1/b_{0}\right)$. Next we combine (Equation 4) using all data with priors to get the full conditional distribution for parameters $\beta^{*}$, $\sigma_{\beta }^{2}$, $b_{1}$, $\sigma_{b1}^{2}$, $b_{2}$, $\sigma_{b2}^{2}\;$ and *r*.

##### Full conditional distributions:

The full conditional distribution of $\;\beta^{*}$ is given as:

$$\;f(\beta^{*}|y,ELSE)∼N\left({\overset{˜}{\beta }}_{0},{\overset{˜}{\Sigma }}_{0}\right)$$ (5)

where ${\tilde{\Sigma }}_{0}={\left(\Sigma_{0}^{-1}\sigma_{\beta }^{-2}+X^{T}D_{\omega }X\right)}^{-1}$, ${\tilde{\beta }}_{0}={\tilde{\Sigma }}_{0}(\Sigma_{0}^{-1}\sigma_{\beta }^{-2}\beta_{0}-X^{T}D_{\omega }\Sigma_{h=1}^{2}Z_{h}b_{h}+X^{T}\kappa )$, $\;y_{ijk}={\left[y_{ijk1},\ldots ,y_{ijkn}\right]}^{T}$, $\;y_{ij}={\left[y_{ij1}^{T},\ldots ,y_{ijK}^{T}\right]}^{T}$, $\;y_{i}={\left[y_{i1}^{T},\ldots ,y_{iJ}^{T}\right]}^{T}$, $y={\left[y_{1}^{T},\ldots ,y_{I}^{T}\right]}^{T},\;\kappa_{ijk}=\frac{1}{2}{\left[y_{ijk1}-r,\ldots ,y_{ijkn}-r\right]}^{T}$, $\;\kappa_{ij}={\left[\kappa_{ij1}^{T},\ldots ,\kappa_{ijK}^{T}\right]}^{T}$, $\;\kappa_{i}={\left[\kappa_{i1}^{T},\ldots ,\kappa_{iJ}^{T}\right]}^{T}$, $\kappa ={\left[\kappa_{1}^{T},\ldots ,\kappa_{I}^{T}\right]}^{T}$, $\;X_{ijk}={\left[1_{n}^{T}⊗x_{ik}\right]}^{T}$, $\;X_{ij}={\left[X_{ij1}^{T},\ldots ,\;X_{ijK}^{T}\right]}^{T}$, $\;X_{i}={\left[X_{i1}^{T},\ldots ,\;X_{iJ}^{T}\right]}^{T}$, $X={\left[X_{1}^{T},\ldots ,\;X_{I}^{T}\right]}^{T}$, $D_{\omega ijk}=diag\left(\omega_{ijk1},\;\ldots ,\;\omega_{ijkn}\right)$, $D_{\omega ij}=diag\left(D_{\omega ij1},\;\ldots ,\;D_{\omega ijK}\right)$, $\;D_{\omega i}=diag\left(D_{\omega i1},\;\ldots ,\;D_{\omega iJ}\right)$, $D_{\omega }=diag\left(D_{\omega 1},\;\ldots ,\;D_{\omega I}\right)$, $b_{1}={\left[b_{11},\ldots ,\;b_{1J}\right]}^{T}$, $b_{2i}={\left[b_{2i1},\ldots ,\;b_{2iJ}\right]}^{T}$, $b_{2}={\left[b_{21}^{T},\ldots ,\;b_{2I}^{T}\right]}^{T}$, $Z_{1i}=\left[\begin{array}{ccc} \begin{array}{cc} 1_{n} & 0\\ 0 & 1_{n} \end{array} & \begin{array}{c} \ldots \\ \ldots \end{array} & \begin{array}{c} 0\\ 0 \end{array}\\ \begin{array}{cc} \vdots & \vdots \end{array} & ֻ & \vdots \\ \begin{array}{cc} 0 & 0 \end{array} & \ldots & 1_{n} \end{array}\right]$, $Z_{1}={\left[Z_{11}^{T},\ldots ,\;Z_{1I}^{T}\right]}^{T}$ and $Z_{2}=Z_{1}*∼X$, where *∼  indicates the horizontal Kronecker product between $Z_{1}$ and X. The horizontal Kronecker product performs a Kronecker product of $Z_{1}$ and ***X***, and creates a new matrix by stacking these row vectors into a matrix. $Z_{1}$ and ***X*** must have the same number of rows, which is also the same number of rows in the result matrix. The number of columns in the result matrix is equal to the product of the number of columns in $Z_{1}$ and ***X***. When the prior for $\;\beta^{*}\propto \;$ constant, the posterior distribution of $\;\beta^{*}$ is also normally distributed, $N\left({\tilde{\beta }}_{0},{\tilde{\Sigma }}_{0}\right)$, but we set the term $\Sigma_{0}^{-1}\sigma_{\beta }^{-2}$ to zero in both ${\tilde{\Sigma }}_{0}$ and $\tilde{\beta }$.

The fully conditional distribution of $\omega_{ijkt}$ is

$$f(\omega_{ijkt}|y,ELSE)∼PG(y_{ijkt}+r,x_{ik}^{T}\;\beta^{*}+b_{1j}+\;b_{2ij})$$ (6)

Defining $\eta^{1}=X\;\beta^{*}+Z_{2}b_{2}$, the conditional distribution of $b_{1}$ is given as

$$f(b_{1}|y,ELSE)∼N\left({\overset{˜}{b}}_{1},\;F_{1}\right)$$ (7)

with $\;F_{1}={\left(\sigma_{b_{1}}^{-2}G_{1}^{-1}+Z_{1}^{T}D_{\omega }Z_{1}\right)}^{-1},\;{\overset{˜}{b}}_{1}=\;F_{1}\left(Z_{1}^{T}\kappa -Z_{1}^{T}D_{\omega }\eta^{1}\right)$. Similarly, by defining $\eta^{2}=X\;\beta^{*}+Z_{1}b_{1}$, the conditional distribution of $b_{2}$ is

$$f(b_{2}|y,ELSE)∼N\left({\overset{˜}{b}}_{2},\;F_{2}\right)$$ (8)

where $\;F_{2}={\left(\sigma_{b_{2}}^{-2}G_{2}^{-1}+Z_{2}^{T}D_{\omega }Z_{2}\right)}^{-1},{\tilde{\;b}}_{2}=\;F_{2}\left(Z_{2}^{T}\kappa -Z_{2}^{T}D_{\omega }\eta^{2}\right)$.

The fully conditional distribution of $\sigma_{b_{h}}^{2}$ for h=1,2,  is

$$f(\sigma_{b_{h}}^{2}|y,ELSE)∼\chi^{-2}({\tilde{\nu }}_{b}=\nu_{b_{h}}+n_{b_{h}},{\tilde{S}}_{b}=(b_{h}^{T}G_{h}^{-1}b_{h}+\nu_{b_{h}}S_{b_{h}})/\nu_{b_{h}}+n_{b_{h}})$$ (9)

with $n_{b_{1}}$=*J* and $n_{b_{2}}$=*IJ*.

The conditional distribution of $\sigma_{\beta^{*}}^{2}$ is

$$f(\sigma_{\beta^{*}}^{2}|y,ELSE)∼\chi^{-2}({\tilde{\nu }}_{\beta^{*}}=\nu_{\beta^{*}}+I+IK,{\tilde{S}}_{\beta }=\frac{\left[{\left(\;\beta^{*}-\beta_{0}\right)}^{T}\Sigma_{0}^{-1}\left(\;\beta^{*}-\beta_{0}\right)+\nu_{\beta^{*}}S_{\beta^{*}}\right]}{{\tilde{\nu }}_{\beta^{*}}})$$ (10)

Taking advantage of the fact that the NB distribution can also be generated using a Poisson representation (Quenouille 1949) as $Y=\overset{L}{\underset{l=1}{\sum }}u_{l},\;\mathrm{where}\;u_{l}∼Log\left(\pi \right),\;$ $\pi =\frac{\mu }{r+\mu }$ and is independent of L∼ Pois[−rlog(1−π)], where Log and Pois denote logarithmic and Poisson distributions, respectively. Then, we infer a latent count *L* for each Y ∼ NB(μ,r) conditional on *Y* and *r*. Therefore, following Zhou *et al.* (2012), we obtain the full conditional of *r* by alternating

$$f(r|y,ELSE)∼G\left(a_{0}-\;\overset{I}{\underset{i=1}{\sum }}\overset{J}{\underset{j=1}{\sum }}\overset{K}{\underset{k=1}{\sum }}\overset{n_{ijk}}{\underset{t=1}{\sum }}\log\left(1-\pi_{ijkt}\right),\;\frac{1}{b_{0}+\sum_{i=1}^{I}\sum_{j=1}^{J}\sum_{k=1}^{K}\sum_{t=1}^{n_{ijk}}L_{ijkt}}\;\;\right)$$ (11)

$$f(L_{ijkt}|y,ELSE)∼\;CRT\left(y_{ijkt},r\right)$$ (12)

where $CRT\left(y_{ijkt},r\right)$ denotes a Chinese restaurant table (CRT) count random variable that can be generated as $L_{ijkt}=\Sigma_{l=1}^{y_{ijkt}}d_{l},$ where $\;d_{l}∼Bernoulli\left(\frac{r}{l-1+r}\right)$. For details of the CRT random variable derivation, see Zhou and Carin (2012, 2015).

#### Gibbs sampler:

The Gibbs sampler for the latent parameters of the NB with G×E  can be implemented by sampling repeatedly from the following loop:

1. Sample $\omega_{ijkt}$ values from the Pólya-Gamma distribution in (6).
2. Sample $L_{ijkt}∼CRT\left(y_{ijkt},r\right)$ from (12).
3. Sample the scale parameter (r) from the gamma distribution in (11).
4. Sample the location effects ($\;\beta^{*})$ from the normal distribution in (5).
5. Sample the random effects $(b_{1})\;\;$ from the normal distribution in (7).
6. Sample the random effects $(b_{2})\;\;$ from the normal distribution in (8).
7. Sample the variance effects ($\sigma_{b_{h}}^{2})$ with h=1,2,  from the scaled inverted $\chi^{2}$ distribution in (9).
8. Sample the variance effect ($\sigma_{\beta^{*}}^{2})$ from the scaled inverted $\chi^{2}$ distribution in (10).
9. Return to step 1 or terminate when chain length is adequate to meet convergence diagnostics.

#### Model implementation:

The Gibbs sampler described above for the BMNB model was implemented in R-Core Team (2015). Implementation was done under a Bayesian approach using Markov Chain Monte Carlo (MCMC) through the Gibbs sampler algorithm, which samples sequentially from the full conditional distribution until it reaches a stationary process, converging with the joint posterior distribution (Gelfand and Smith 1990). To decrease the potential impact of MCMC errors on prediction accuracy, we performed a total of 60,000 iterations, with a burn-in of 30,000, so that 30,000 samples were used for inference. We did not apply thinning of the chains following the suggestions of Geyer (1992), MacEachern and Berliner (1994), and Link and Eaton (2012), who provide justification of the ban on subsampling MCMC output for approximating simple features of the target distribution (*e.g.*, means, variances, and percentiles). We implemented the prior specification given in the section *Bayesian mixed negative binomial regression* with $\;\beta^{*}|\sigma_{\beta }^{2}∼N_{p}\left(\beta_{0}=0_{9}^{T},I_{9}\times 10,000\right),\;\;b_{1}|\sigma_{b1}^{2}∼N_{nb1}\left(0_{nb1}^{T},G_{1}\sigma_{b1}^{2}\right)$, where $G_{1\;}$ is the GRM, that is, the covariance matrix of the random effects, $\sigma_{b1}^{2}∼\;\chi^{-2}\left(\nu_{b1}=3,S_{b1}=0.001\right),$ $b_{2}|\sigma_{b2}^{2}∼N_{nb2}\left(0_{nb2}^{T},G_{2}\sigma_{b2}^{2}\right)$, $G_{2\;}$ is the covariance matrix of the random effects that belong to the G×E term, $\sigma_{b2}^{2}\;∼\;\chi^{-2}\left(\nu_{b2}=3,S_{b2}=0.001\right),$ and $r∼G\left(a_{0}=0.01,1/(b_{0}=0.01)\right).$ All these hyper-parameters were chosen to lead weakly informative priors. The convergence of the MCMC chains was monitored using trace plots and autocorrelation functions. We also conducted a sensitivity analysis on the use of the inverse gamma priors for the variance components, and we observed that the results are robust under different choices of priors.

#### Assessing prediction accuracy:

We used cross-validation to compare the prediction accuracy of the proposed models for count phenotypes. We implemented a 10-fold cross-validation, that is, the data set was divided into 10 mutually exclusive subsets; each time we used nine subsets for the training set, and the remaining one for the validation set. The training set was used to fit the model, and the validation set was used to evaluate the prediction accuracy of the proposed models. To compare the prediction accuracy of the proposed models, we calculated the Spearman correlation (Cor) and the mean square error of prediction (MSEP), both calculated using the observed and predicted response variables of the validation set. Models with large values of Cor indicate better prediction accuracy, while small MSEP indicate better prediction performance. The predicted observations, ${\hat{y}}_{ijkt}$, were calculated with *M* collected Gibbs samples after discarding those of the burn-in period. For **Models NB** and **Pois,** the predicted values were calculated as ${\hat{y}}_{ijkt}=\frac{\sum_{s=1}^{M}\exp(x_{ik}^{T}\;{\hat{\beta }}^{*(s)}+\log\left({\hat{r}}^{\left(s\right)}\right)+{\hat{g}}_{j}{}^{\left(s\right)}+{\hat{g}E}_{ij}{}^{\left(s\right)})}{M}$, where ${\hat{r}}^{\left(s\right)},\;\;{\hat{\beta }}^{*(s)}$, ${\hat{g}}_{j}{}^{\left(s\right)}\;$, and ${\hat{g}E}_{ij}{}^{\left(s\right)}\;$ are estimates of $\;\beta^{*},$ r,  $g_{j}$, and $gE_{ij}$, for line *j*, block *k*, in environment *i* obtained in the s*th* collected sample. For **Model Normal** as ${\hat{y}}_{ijkt}=\frac{\sum_{s=1}^{M}(x_{ik}^{T}\;{\hat{\beta }}^{\left(s\right)}+{\hat{g}}_{j}{}^{\left(s\right)}+{\hat{g}E}_{ij}{}^{\left(s\right)})}{M}$, and for **Model LN**, the predicted observations were calculated as ${\hat{y}}_{ijkt}=\frac{\sum_{s=1}^{M}\exp(x_{ik}^{T}\;{\hat{\beta }}^{\left(s\right)}+{\hat{g}}_{j}{}^{\left(s\right)}+{\hat{g}E}_{ij}{}^{\left(s\right)}+\frac{{\hat{\sigma }}_{e}^{2\left(s\right)}}{2})}{M}-1$, using the corresponding estimates of each model.

#### Simulation study:

To show the performance of the proposed Gibbs sampler for count phenotypes that takes into account G×E, we performed a simulation study under model (1) with the following linear predictor: $\eta_{ij}=E_{i}+g_{j}+gE_{ij},\;\;$ with two scenarios (S1 and S2). Scenario 1 had three environments (I=3), 20 genotypes (J=20), $G_{1}=I_{20}$, $G_{2}=I_{I}⊗G_{1}\;$ and $\sigma_{b_{1}}^{2}=\sigma_{b_{2}}^{2}=0.5,$ with four different numbers of replicates of each genotype in each environment, n= 5, 10, 20, and 40. Scenario 2 is equal to scenario 1, except that $G_{1}=0.7I_{20}+0.3J_{20}$, where $J_{20}\;$ is a square matrix of ones of the order 20×20. In this second scenario, we imitated the correlation between lines of real data available in genomic selection. The priors used for the simulation study in both scenarios (S1 and S2) were approximately flat for all parameters: for $\beta |\sigma_{\beta }^{2}∼N(\beta_{0}^{T}=[0,0,0],I_{3}\times 10,000)$, for r∼G(0.001,1/0.001), for $\sigma_{b_{1}}^{2}\;$ and $\sigma_{b_{2}}^{2}$ a $\chi^{-2}(0.50002,4.0002)$, while for $b_{1}|\sigma_{b1}^{2}∼N\left(0,G_{1}\sigma_{b1}^{2}\right)$, and for $b_{2}|\sigma_{b2}^{2}∼N\left(0,G_{2}\sigma_{b2}^{2}\right)$. We computed 20,000 MCMC samples; Bayes estimates were computed with 10,000 samples, since the first 10,000 were discarded as burn-in. We report average estimates obtained by using the proposed Gibbs sampler, along with SD (Table 1). All the results in Table 1 are based on 50 replications.

**Table 1:** Posterior mean and posterior SD of the Bayesian method with four sample sizes (n) for Model NB

			n=5		n=10		n=20		n=40	
Scenario	Parameter	True	Mean	SD	Mean	SD	Mean	SD	Mean	SD
	${\text{β}}_{0}$	1.5	1.48	0.36	1.49	0.27	1.54	0.23	1.55	0.21
	${\text{β}}_{1}$	−1	−0.98	0.26	−0.99	0.25	−1.08	0.25	−1.02	0.19
1	${\text{β}}_{2}$	1	1.00	0.27	0.99	0.22	0.99	0.27	0.95	0.22
	*r*	5	5.08	0.92	5.08	0.52	5.02	0.47	5.03	0.33
	${\text{σ}}_{1}^{2}$	0.5	0.54	0.20	0.59	0.18	0.58	0.18	0.59	0.22
	${\text{σ}}_{2}^{2}$	0.5	0.50	0.13	0.52	0.14	0.53	0.11	0.51	0.11
	${\text{β}}_{0}$	1.5	1.48	0.50	1.46	0.50	1.56	0.61	1.47	0.50
	${\text{β}}_{1}$	−1	−1.06	0.23	−1.00	0.20	−1.01	0.22	−1.03	0.19
2	${\text{β}}_{2}$	1	0.95	0.24	1.03	0.22	0.99	0.20	0.97	0.20
	*r*	5	5.10	0.81	4.99	0.59	5.04	0.35	5.03	0.20
	${\text{σ}}_{1}^{2}$	0.5	0.54	0.18	0.57	0.22	0.58	0.19	0.53	0.18
	${\text{σ}}_{2}^{2}$	0.5	0.50	0.12	0.51	0.14	0.53	0.13	0.51	0.10

## Results

Table 1 list the results of the simulation study of both scenarios (S1 and S2). The bias when estimating the parameters is a little larger in S1 compared to S2. Also, parameter ${\text{β}}_{0}$ is the parameter with larger bias (underestimated). Both variances (${\text{σ}}_{1}^{2}$, ${\text{σ}}_{2}^{2}$) are overestimated in scenario 1, but only ${\text{σ}}_{1}^{2}$ is overestimated in scenario 2. Also, with a sample size of n=5, parameter *r* had a larger SD; however, for larger sample sizes (n=20,40), the SD were considerably reduced. In general, there was not a large reduction in SD when the sample size increased from 5 to 10, 20, and 40, the exception being the estimation of *r* in both scenarios, and the estimation of ${\text{β}}_{0}$ in S1, where there was a large reduction in SD when the sample size increased. Although estimations do not totally agree with the true values of the parameters, the proposed Gibbs sampler for count data, which takes into account G×E, did a good job of estimating the parameters, since the estimates are close to the true values with a SD of reasonable size.

In all the experiments (environments) using the real data set, the genotypes were arranged in a randomized complete block design with two blocks; thus the linear predictor used was that given in Equation (1). Using the real data set, we compared four scenarios (S1–S4, given in Table 2) for each model. Table 2 shows that, in the linear predictor, S1 and S2 do not take into account interaction effects between genotypes and environments, only the main effects of these factors. Also, S1 and S3 do not use marker information. These four scenarios were studied to investigate the gain in model fit and prediction ability taking into account the interaction effects, and using the marker information available.

**Table 2:** Scenarios proposed to fit the real data set with Models NB, Pois, Normal and LN

Scenario	Main Effects			Nested Effect	Interaction Effects	
	E	L	G	R(E)	EL	EG
S1	X	X		X		
S2	X		X	X		
S3	X	X		X	X	
S4	X		X	X		X

E, Environment; R, blocks; L, lines; G, lines taking into account markers; EL and EG, interaction effects of E and L, and E and G; R(E) blocks nested in the environment.

The posterior means (Mean), posterior SD of the scalar parameters, and posterior predictive checks for each scenario of the proposed models are given in Table 3. For the four models, the posterior means of the beta regression coefficients, variance components, and overdispersion parameters (*r*) are similar between S1 and S2, and between S3 and S4. In terms of goodness-of-fit measured by the loglikelihood posterior mean (Loglik), the scenarios rank as follows: S3, rank 1; S4, rank 2; S1, rank 3; and S2, rank 4, for the four proposed models, with the exception of **Model Pois**, where the ranking was S3, rank 1; S4, rank 2; S2, rank 3; and S1, rank 4. Therefore, there is evidence that, with the four proposed models in terms of goodness-of-fit, the best scenario is S3. Of the four models under study, Table 3 shows that **Model LN** reports the best fit since it has the largest Loglik.

**Table 3:** Estimated beta coefficients, variance components, and posterior predictive checks for the four scenarios (S1, S2, S3, S4) for each proposed model

	S1		S2		S3		S4	
Parameter	Mean	SD	Mean	SD	Mean	SD	Mean	SD
**Model NB**								
${\text{β}}_{1}^{*}$	−0.93	0.60	−1.05	0.61	−2.52	0.71	−2.38	0.99
${\text{β}}_{2}^{*}$	−0.83	0.71	−1.16	0.66	−2.27	0.58	−2.73	1.00
${\text{β}}_{3}^{*}$	−0.03	0.48	−0.15	0.56	−1.69	0.85	−1.96	0.78
${\text{β}}_{11}$	−0.09	0.52	−0.06	0.65	−0.02	0.54	−0.25	0.67
${\text{β}}_{12}$	0.05	0.51	0.08	0.60	0.10	0.53	−0.13	0.66
${\text{β}}_{21}$	−0.20	0.62	0.05	0.70	−0.27	0.47	0.09	0.67
${\text{β}}_{22}$	−0.05	0.61	0.20	0.66	−0.15	0.46	0.21	0.65
${\text{β}}_{31}$	0.07	0.42	0.11	0.61	0.11	0.61	0.32	0.50
${\text{β}}_{32}$	−0.14	0.41	−0.10	0.59	−0.10	0.60	0.11	0.48
${\text{σ}}_{1}^{2}$	0.43	0.05	1.37	0.17	0.34	0.05	1.03	0.15
${\text{σ}}_{2}^{2}$	–	–	–	–	0.38	0.03	1.04	0.10
*r*	2.80	0.12	2.81	0.12	11.87	1.12	11.55	1.17
Loglik	−1526.65		−1526.88		−1268.83		−1275.25	
Cor	0.69		0.69		0.90		0.89	
MSEP	2.13		2.12		0.75		0.77	
**Model Pois**								
${\text{β}}_{1}^{*}$	−7.14	0.22	−7.21	0.39	−6.69	0.11	−6.80	0.33
${\text{β}}_{2}^{*}$	−7.08	0.13	−7.17	0.11	−7.07	0.16	−7.27	0.19
${\text{β}}_{3}^{*}$	−5.97	0.43	−6.46	0.29	−5.88	0.16	−6.66	0.28
${\text{β}}_{11}$	0.12	0.17	0.07	0.29	−0.25	0.11	−0.34	0.23
${\text{β}}_{12}$	0.27	0.17	0.23	0.29	−0.13	0.11	−0.22	0.23
${\text{β}}_{21}$	0.06	0.14	0.03	0.15	0.14	0.15	0.13	0.17
${\text{β}}_{22}$	0.22	0.14	0.18	0.15	0.25	0.15	0.24	0.17
${\text{β}}_{31}$	0.04	0.34	0.41	0.21	−0.09	0.13	0.51	0.19
${\text{β}}_{32}$	−0.20	0.33	0.17	0.21	−0.31	0.13	0.28	0.19
${\text{σ}}_{1}^{2}$	0.44	0.05	1.46	0.17	0.35	0.05	1.03	0.14
${\text{σ}}_{2}^{2}$	–	–	–	–	0.38	0.03	1.05	
*r*	1000.00		1000.00		1000.00		1000.00	
Loglik	−1477.63		−1477.52		−1228.73		−1234.97	
Cor	0.66		0.66		0.90		0.89	
MSEP	1.87		1.86		0.74		0.76	
**Model Normal**								
${\text{β}}_{1}$	−12.30	5.86	7.90	4.36	13.70	3.69	9.22	3.11
${\text{β}}_{2}$	−12.20	5.80	7.93	4.41	13.60	3.73	9.11	3.16
${\text{β}}_{3}$	−10.40	5.87	9.66	4.36	15.50	3.69	10.94	3.10
${\text{σ}}_{1}^{2}$	0.96	0.16	1.42	0.35	0.72	0.18	1.58	0.40
${\text{σ}}_{2}^{2}$	–	–	–	–	1.33	0.18	1.13	0.34
*r*	2.75	0.14	2.91	0.15	1.67	0.11	2.23	0.17
Loglik	−1918.00		−1957.00		−1542.00		−1747.00	
Cor	0.60		0.56		0.83		0.71	
MSEP	2.41		2.60		1.07		1.68	
**Model LN**								
${\text{β}}_{1}$	−3.95	0.51	−6.34	3.33	1.41	0.48	3.32	1.31
${\text{β}}_{2}$	−3.95	0.48	−6.33	3.32	1.41	0.49	3.32	1.29
${\text{β}}_{3}$	−3.51	0.49	−5.85	3.33	1.86	0.49	3.79	1.31
${\text{σ}}_{1}^{2}$	0.09	0.01	0.15	0.03	0.07	0.01	0.16	0.03
${\text{σ}}_{2}^{2}$	–	–	–	–	0.08	0.01	0.05	0.02
*r*	0.17	0.01	0.181	0.009	0.11	0.01	0.15	0.01
Loglik	−484.00		−518.00		−125.00		−354.00	
Cor	0.71		0.68		0.88		0.79	
MSEP	2.50		2.63		1.25		1.97	

The beta coefficients corresponding to effects of environments (β_1_, β_2_, β_3_) are given for models Normal and LN only. Mean, posterior mean; SD posterior SD.

Table 4 presents the mean and SD of the posterior predictive checks (Cor and MSEP) for each location (Batan 2012, Batan 2014, and Ecuador 2014) resulting from the 10-fold cross-validation implemented for the four models and four scenarios. The predictive checks given in Table 4 were calculated using the testing set. In **Model NB**, according to the Spearman correlation, the ranking of scenarios was as follows: in Batan 2012, 1 for S4, 2 for S3, 3 for S1, and 4 for S2. In Batan 2014, the ranking was 1 for S4, 2 for S3 and 3 for S1 and S2. In Ecuador 2014, the ranking was 1 S3, 2 for S2, 3 for S1, and 4 for S4. With the MSEP, the ranking for **Model NB** in Batan 2012 was 1 for S3, 2 for S4, 3 for S1 and 4 for S2. In Batan 2014, the ranking was 1 for S2, 2 for S1, 3 for S3, and 4 for S4. In Ecuador 2014, the ranking in terms of MSEP was 1 for S3, 2 for S2, 3 for S4, and 4 for S1. Under **Model Pois,** the ranking of the four scenarios in each locality was exactly the same as the ranking reported for **Model NB**. For **Model Normal** in terms of the Spearman correlation, S1 was the best in prediction accuracy in Batan 2012, while scenario 4 was the worst in all three locations. In terms of MSEP, the best scenario was S3 in Batan 2012 and Ecuador 2014, and the worst was S4 in Batan 2014 and Ecuador 2014. For **Model LN** in terms of the Spearman correlation, the best scenarios were scenarios S1, S2 and S3 and the worst was S4 in Batan 2012. In Batan 2014, the best scenario was S1, then scenario S3 and the worst was scenario S4. In Ecuador 2014, the best scenario was scenario S1 and S3, then S2 and S4. In terms of MSEP for Batan 2012, the best scenario was S3, then S1 and S2 and the worst was S4. In Batan 2014, the best scenario was S1, then S2 and the worst was scenario S4. Finally, in Ecuador 2014, the best scenario was S3, then S2 and the worst was scenario S1.

**Table 4:** Estimated posterior predictive checks with cross-validation for Models NB, Pois, Normal and LN

		Batan 2012		Batan 2014		Ecuador 2014	
Scenario		Cor	MSEP	Cor	MSEP	Cor	MSEP
**Model NB**							
S1	Mean	0.43 (3)	0.98 (3.5)	0.43 (3.5)	1.39 (2)	0.18 (3)	11.733 (4)
	SD	0.33	0.72	0.33	1.35	0.40	9.471
S2	Mean	0.42 (4)	0.98 (3.5)	0.43 (3.5)	1.38 (1)	0.20 (2)	11.222 (2)
	SD	0.33	0.72	0.33	1.36	0.37	8.614
S3	Mean	0.54 (2)	0.49 (1)	0.52 (2)	1.48 (3)	0.22 (1)	8.645 (1)
	SD	0.28	0.38	0.29	2.32	0.39	5.688
S4	Mean	0.56 (1)	0.61 (2)	0.56 (1)	1.85 (4)	0.12 (4)	11.343 (3)
	SD	0.24	0.44	0.22	2.68	0.41	8.154
**Model Pois**							
S1	Mean	0.43 (3)	0.98 (3.5)	0.43 (3.5)	1.39 (2)	0.18 (3)	11.733 (4)
	SD	0.33	0.72	0.33	1.35	0.40	9.471
S2	Mean	0.42 (4)	0.98 (3.5)	0.43 (3.5)	1.38 (1)	0.20 (2)	11.222 (2)
	SD	0.33	0.72	0.33	1.36	0.37	8.614
S3	Mean	0.54 (2)	0.48 (1)	0.52 (2)	1.48 (3)	0.22 (1)	8.645 (1)
	SD	0.28	0.38	0.29	2.32	0.39	5.688
S4	Mean	0.56 (1)	0.61 (2)	0.56 (1)	1.85 (4)	0.12 (4)	11.343 (3)
	SD	0.24	0.44	0.22	2.68	0.41	8.154
**Model Normal**							
S1	Mean	0.36(1)	1.10 (4)	0.37 (1.5)	1.79 (1)	0.15 (1.5)	7.425 (2)
	SD	0.28	0.88	0.39	1.70	0.32	4.151
S2	Mean	0.34 (2)	0.99 (2)	0.33 (3)	2.01 (3)	0.07 (3)	7.454 (3)
	SD	0.33	0.65	0.44	2.46	0.33	4.339
S3	Mean	0.33 (3)	0.81 (1)	0.37 (1.5)	1.96 (2)	0.15 (1.5)	7.318 (1)
	SD	0.30	0.46	0.40	2.99	0.29	4.159
S4	Mean	0.27 (4)	1.03 (3)	0.24 (4)	2.37 (4)	0.04 (4)	8.482 (4)
	SD	0.34	0.73	0.45	3.42	0.24	4.326
**Model LN**							
S1	Mean	0.51 (2)	0.66 (2.5)	0.46 (1)	1.60 (1)	0.15 (1.5)	8.10 (4)
	SD	0.21	0.42	0.31	2.35	0.38	5.11
S2	Mean	0.51 (2)	0.66 (2.5)	0.43 (3.5)	1.78 (2)	0.09 (3.5)	7.82 (2)
	SD	0.22	0.39	0.35	2.82	0.46	5.31
S3	Mean	0.51 (2)	0.64 (1)	0.45 (2)	1.871 (3)	0.15 (1.5)	7.76 (1)
	SD	0.21	0.45	0.31	3.16	0.37	5.21
S4	Mean	0.43 (4)	0.72 (4)	0.43 (3.5)	1.95 (4)	0.09 (3.5)	8.04(3)
	SD	0.25	0.42	0.33	3.15	0.41	5.18

The numbers in parentheses denote the ranking of the four scenarios for each posterior predictive check.

Table 5 gives the average of the ranks of the two posterior predictive checks (Cor and MSEP) that were used. Since we are comparing four scenarios for each model, the values of the ranks range from 1 to 4, and the lower the values, the better the scenario. For ties, we assigned the average of the ranges that would have been assigned had there been no ties. Table 5 shows that the best scenarios were S3 and S4 under **Models NB** and **Pois** in Batan 2012. In Batan 2014, under **Models NB** and **Pois**, the best scenario was S2, while in Ecuador 2014, the best scenarios were S3. Under **Model Normal**, the best scenario was S1 in Batan 2014 S1 and S3 in Ecuador 2014, while in Batan 2012, the best scenarios were S2 and S3. Finally, under **Model LN**, the best scenario was S3 in Ecuador 2014, S3 in Batan 2012 and S1 in Batan 2014.

**Table 5:** Rank averages for the four scenarios for each model resulting from the 10-fold cross-validation implemented

Scenario	Batan 2012	Batan 2014	Ecuador 2014	Batan 2012	Batan 2014	Ecuador 2014
	**Model NB**			**Model Normal**		
S1	3.25	2.75	3.5	2.5	1.25	1.75
S2	3.75	2.25	2	2	3	3
S3	1.5	2.5	1	2	1.75	1.75
S4	1.5	2.5	3.5	3.5	4	4
	**Model Pois**			**Model LN**		
S1	3.25	2.75	3.5	2.25	1	2.75
S2	3.75	2.25	2	2.25	2.75	2.75
S3	1.5	2.5	1	1.5	2.5	1.25
S4	1.5	2.5	3.5	4	3.75	3.25

Each average was obtained as the mean of the rankings given in Table 4 for the two posterior predictive checks (Cor and MSEP) in each scenario.

Results in Table 4 and Table 5 indicate that the best models, in terms of prediction accuracy, are **Models NB** and **Pois**, since they had better predictions in the validation set based on both posterior predictive checks (Cor and MSEP) implemented, although, in terms of goodness-of-fit, **Model LN** was the best. These results are in partial agreement with the findings of Montesinos-Lopez *et al.* (2015c), who came to the conclusion that **Models NB** and **Pois** are good alternatives for modeling count data, although in this study, the best predictions were produced by **Model LN**. However, this model did not take into account G×E interaction.

## Discussion

Generalized linear mixed models (GLMM) are widely recognized as one of the major methodological developments of the second half of the twentieth century. The main factor contributing to the success of their wide applicability over the last 30 years or so has been their flexibility, since they can be applied to many different types of data (Berridge and Crouchley 2011). These types of data include continuous interval/scale, categorical (including binary and ordinal) data, count data, beta data, and others. Each member of the GLMM family is appropriate for a specific type of data (Berridge and Crouchley 2011). However, GLMM for non-normal data are scarce in the context of genome-enabled prediction, since most of the models developed so far are linear mixed models (mixed models for Gaussian data). For this reason, we believe that developing specific methods for count data for genome-enabled prediction can help to improve the selection of candidate genotypes early when the phenotypes are counts. Because using transformation to approximate the counts to normality, or assuming that the counts are normally distributed, frequently produces poor parameter estimates and lower power. Also, parameter interpretation is more difficult when transformation is used (Stroup 2015). However, in genomic selection, phenotypic data (dependent variable) are not currently taken into account before deciding on the modeling approach to be used, mainly due to the lack of genome-enabled prediction models for non-normal phenotypes. Although our proposed Bayesian regression models are only for count data, they help fill this lack of genome-enabled prediction models for non-normal data.

Another advantage of our proposed methods for count data is that they take into account the nonlinear relationship between responses, and consider the specific properties of counts, including discreteness, non-negativity, and overdispersion (variance greater than the mean); this guarantees that the predictive response will not be negative, which makes no sense for count data. In addition, our methods help modeling G×E for count data in the context of genome-enabled prediction, which plays a central role in plant breeding for the selection of candidate genotypes that present high stability over a wide range of environmental conditions, and for the prediction of yet-to-be observed phenotypes when the relative performance of genotypes varies across environments.

Another advantage of our proposed method is that the proposed Gibbs sampler has an analytical solution because we were able to obtain all the analytically required full conditional distributions. This is important, because, of all the computational intensive methods for fitting complex multilevel models, the Gibbs sampler is the most popular due to its simplicity and ability to effectively generate samples from high-dimensional probability distributions (Park and van Dyk 2009). This was possible because we constructed our Gibbs sampler using the data augmentation approach proposed by Polson *et al.* (2013). For this reason, we believe it is an attractive alternative for fitting complex count data that arise in the context of genomic selection.

Our proposed methods showed superior performance in terms of prediction accuracy compared to **Models Normal** and **LN**. Also, we observed that, in **Models NB** and **Pois**, taking into account G×E considerably increased the prediction accuracy, which was expected since there is enough scientific evidence that including G×E interaction improves prediction accuracy. However, to use these models correctly, it is important to first understand the types of data we have before deciding on the modeling approach to be used. If the phenotypic data are normally distributed, the linear mixed models for genome-enabled prediction developed so far for Gaussian phenotypes should be used. If the phenotypic data are binary or categorical ordinal, the methods proposed by Montesinos-López *et al.* (2015a, 2015b) developed for ordinal data for genome-enabled prediction are preferred. If the phenotypic data are counts (number of panicles per plant, number of seeds per panicle, weed count per plot, etc.), and the counts are small, the models developed in this study, and those proposed by Montesinos-López *et al.* (2015c), are the best option, since they have more advantages over the conventional linear mixed models with Gaussian response, as was observed when we applied them to the real data set. We also need to keep in mind that **Model Pois** will be enough when the equi-dispersion (equality of mean and variance) is supported by the data at hand. However, when this assumption is violated, and the variance of the counts exceeds the mean count, overdispersion is present; in this situation, the most appropriate model is the NB model because it can control the overdispersion with the scale parameter (r), and improve parameter estimates, power, and predictions (Yaacob *et al.* 2010). Finally, more research is needed to study the proposed methods using other real data sets, and extend the proposed genomic-enabled prediction models to deal with the large number of zeros in count response variables, and for modeling multiple traits.

## Appendix A

### Derivation of full conditional distribution for all parameters.

#### Full conditional for $\;\beta^{*}$

$$f\left(\;\beta^{*}|y,ELSE\right)=\;\overset{I}{\underset{i=1}{\prod }}\overset{J}{\underset{j=1}{\prod }}\overset{K}{\underset{k=1}{\prod }}\overset{n_{ijk}}{\underset{t=1}{\prod }}\mathrm{Pr}\left(Y_{ijkt}=y_{ijkt}|x_{ik}^{T},r,\omega_{ijkt},b_{1i},b_{2ij}\right)f\left(\;\beta^{*}\right)$$

$$\propto \exp\left(\kappa^{T}X\;\beta^{*}+\kappa^{T}\overset{2}{\underset{h=1}{\sum }}Z_{h}b_{h}-\frac{1}{2}{\left(X\;\beta^{*}+\overset{2}{\underset{h=1}{\sum }}Z_{h}b_{h}\right)}^{T}D_{\omega }\left(X\;\beta^{*}+\overset{2}{\underset{h=1}{\sum }}Z_{h}b_{h}\right)-\frac{1}{2}{\left(\;\beta^{*}-\beta_{0}\right)}^{T}\Sigma_{0}^{-1}\sigma_{\beta }^{-2}(\;\beta^{*}-\beta_{0})\right)$$

$$\propto \exp\left(-\frac{1}{2}[\beta^{*T}\left(\Sigma_{0}^{-1}\sigma_{\beta }^{-2}+X^{T}D_{\omega }X\right)\;\beta^{*}-2{\left(\Sigma_{0}^{-1}\sigma_{\beta }^{-2}\beta_{0}-X^{T}D_{\omega }\overset{2}{\underset{h=1}{\sum }}Z_{h}b_{h}+X^{T}\kappa \right)}^{T}\;\beta^{*}]\right)$$

$$\propto \exp\left(-\frac{1}{2}[{\left(\;\beta^{*}-{\tilde{\beta }}_{0}\right)}^{T}{\tilde{\Sigma }}_{0}^{-1}(\;\beta^{*}-{\tilde{\beta }}_{0})]\right)\propto N\left({\tilde{\beta }}_{0},{\tilde{\Sigma }}_{0}\right)$$

where ${\tilde{\Sigma }}_{0}={\left(\Sigma_{0}^{-1}\sigma_{\beta }^{-2}+X^{T}D_{\omega }X\right)}^{-1},$ ${\tilde{\beta }}_{0}={\tilde{\Sigma }}_{0}\left(\Sigma_{0}^{-1}\sigma_{\beta }^{-2}\beta_{0}-X^{T}D_{\omega }\overset{2}{\underset{h=1}{\sum }}Z_{h}b_{h}+X^{T}\kappa \right)$.

#### Full conditional for $\omega_{\mathrm{ijkt}}$

$$f\left(\omega_{ijkt}|y,ELSE\right)\propto \exp\left[-\frac{\omega_{ijkt}{\left(x_{ik}^{T}\;\beta^{*}+b_{1i}+b_{2ij}\right)}^{2}}{2}\right]f(\omega_{ijkt};y_{ijkt}+r,0)\propto \exp\left[-\frac{\omega_{ijkt}{\left(x_{ik}^{T}\;\beta^{*}+b_{1i}+b_{2ij}\right)}^{2}}{2}\right]f(\omega_{ijkt};y_{ijkt}+r,0)\;\propto \;PG(y_{ijkt}+r,x_{ik}^{T}\;\beta^{*}+b_{1i}+b_{2ij})$$

#### Full conditional for $b_{1}$*:*

Defining $\eta^{1}=X\;\beta^{*}+Z_{2}b_{2}$ the conditional distribution of $b_{1}$ is given as

$$f\left(b_{1}|y,ELSE\right)\propto \exp\left(\kappa^{T}Z_{1}b_{1}-\frac{1}{2}{\left(Z_{1}b_{1}+\eta^{1}\right)}^{T}D_{\omega }\left(Z_{1}b_{1}+\eta^{1}\right)\right)\;f(b_{1}|\sigma_{b_{1}}^{2})$$

$$\propto \exp\left\{-\frac{1}{2}\;\left[b_{1}^{T}\left(\sigma_{b_{1}}^{-2}G_{1}^{-1}+Z_{1}^{T}D_{\omega }Z_{1}\right)b_{1}-2\;{\left(Z_{1}^{T}\kappa -Z_{1}^{T}D_{\omega }\eta^{1}\right)}^{T}\;b_{1}\right]\right\}$$

$$\propto \exp\left\{-\frac{1}{2}\;{\left(b_{1}-{\tilde{b}}_{1}\right)}^{T}F_{1}^{-1}(b_{1}-{\tilde{b}}_{1})\right\}∼N\left({\tilde{b}}_{1},\;F_{1}\right)$$

where$\;F_{1}={\left(\sigma_{b_{1}}^{-2}G_{1}^{-1}+Z_{1}^{T}D_{\omega }Z_{1}\right)}^{-1}\;\mathrm{and}\;{\tilde{b}}_{1}=\;F_{1}\left(Z_{1}^{T}\kappa -Z_{1}^{T}D_{\omega }\eta^{1}\right)$.

#### Full conditional for $b_{2}$*:*

Defining $\eta^{2}=X\;\beta^{*}+Z_{1}b_{1}$, the conditional distribution of $b_{2}$ is given as

$$f\left(b_{2}\text{|}y,\text{ELSE}\right)\propto \exp\left(\kappa^{T}Z_{2}b_{2}-\frac{1}{2}{\left(Z_{2}b_{2}+\eta^{2}\right)}^{T}D_{\omega }\left(Z_{2}b_{2}+\eta^{2}\right)\right)\;f(b_{2}|\sigma_{b_{2}}^{2})$$

$$\propto \exp\left\{-\frac{1}{2}\;\left[b_{2}^{T}\left(\sigma_{b_{2}}^{-2}G_{2}^{-1}+Z_{2}^{T}D_{\omega }Z_{2}\right)b_{2}-2\;{\left(Z_{2}^{T}\kappa -Z_{2}^{T}D_{\omega }\eta^{2}\right)}^{T}\;b_{2}\right]\right\}$$

$$\propto \exp\left\{-\frac{1}{2}\;{\left(b_{2}-{\tilde{b}}_{2}\right)}^{T}F_{2}^{-1}(b_{2}-{\tilde{b}}_{2})\right\}∼N\left({\tilde{b}}_{2},\;F_{2}\right)$$

where$\;F_{2}={\left(\sigma_{b_{2}}^{-2}G_{2}^{-1}+Z_{2}^{T}D_{\omega }Z_{2}\right)}^{-1}\;\mathrm{and}\;{\tilde{b}}_{2}=\;F_{2}\left(Z_{2}^{T}\kappa -Z_{2}^{T}D_{\omega }\eta^{2}\right)$.

#### Full conditional for $\sigma_{b_{h}}^{2}$

$$f\left(\sigma_{b_{h}}^{2}|y,\text{ELSE}\right)\propto \frac{1}{{\left(\sigma_{b_{h}}^{2}\right)}^{\frac{\nu_{b_{h}}+n_{b_{h}}}{2}+1}}\exp\left(-\frac{b_{h}^{T}G_{h}^{-1}b_{h}+\nu_{b_{h}}S_{b_{h}}}{2\sigma_{b_{h}}^{2}}\right)$$

$$\propto \chi^{-2}({\tilde{\nu }}_{b}=\nu_{b_{h}}+n_{b_{h}},{\tilde{S}}_{b}=(b_{h}^{T}G_{h}^{-1}b_{h}+\nu_{b_{h}}S_{b_{h}})/\nu_{b_{h}}+n_{b_{h}})$$

with $n_{b_{1}}=$ J, $n_{b_{2}}=$ IJ and h=1,2.

#### Full conditional for $\sigma_{\beta^{*}}^{2}$

$$f\left(\sigma_{\beta^{*}}^{2}|y,ELSE\right)\propto \frac{1}{{\left(\sigma_{\beta^{*}}^{2}\right)}^{\frac{\nu_{\beta^{*}}+I+IK}{2}+1}}\exp\left(-\frac{{\left(\;\beta^{*}-\beta_{0}\right)}^{T}\Sigma_{0}^{-1}(\;\beta^{*}-\beta_{0})+\nu_{\beta^{*}}S_{\beta^{*}}}{2\sigma_{\beta^{*}}^{2}}\right)$$

$$\propto \chi^{-2}\left({\tilde{\nu }}_{\beta^{*}}=\nu_{\beta^{*}}+I+IK,{\tilde{S}}_{\beta }=\frac{\left[{\left(\;\beta^{*}-\beta_{0}\right)}^{T}\Sigma_{0}^{-1}\left(\;\beta^{*}-\beta_{0}\right)+\nu_{\beta^{*}}S_{\beta^{*}}\right]}{{\tilde{\nu }}_{\beta^{*}}}\right)$$

#### Full conditional for *r*

To make the inference of *r*, we first place a gamma prior on it as $r∼\text{G}\left({\text{a}}_{0},1/{\text{b}}_{0}\right)$. Then we infer a latent count L for each count conditional on *Y* and *r*. To derive the full conditional of *r*, we use the following parameterization of the NB distribution: Y ∼ NB(π,r) with $\text{π}=\frac{\text{μ}}{r+\text{μ}}$. Since L∼ Pois[−rlog(1−π)], by construction we can use the Gamma-Poisson conjugacy to update *r*. Therefore,

$$f\left(r|y,ELSE\right)\propto f(r)\overset{I}{\underset{i=1}{\prod }}\overset{J}{\underset{j=1}{\prod }}\overset{K}{\underset{k=1}{\prod }}\overset{n_{ijk}}{\underset{t}{\prod }}f\left(y_{ijkt}|L_{ijkt}\right)f(L_{ijkt})$$

$$\propto \;r^{a_{0}-1}\exp\left(-rb_{0}\right)\overset{I}{\underset{i=1}{\prod }}\overset{J}{\underset{j=1}{\prod }}\overset{K}{\underset{k=1}{\prod }}\overset{n_{ijk}}{\underset{t}{\prod }}[-r\log(1-\pi_{ijk})]{\;}^{L_{ijkt}}\exp[r\;\log(1-\pi_{ijk})]\;$$

$$\;\;\;\;\;\;\;\;\;\;\;\;\;\propto \;r^{a_{0}+\overset{I}{\underset{i=1}{\sum }}\overset{J}{\underset{j=1}{\sum }}\overset{K}{\underset{k=1}{\sum }}\overset{n_{ijk}}{\underset{t=1}{\sum }}L_{ijt}-1}\exp[-(b_{0}-\overset{I}{\underset{i=1}{\sum }}\overset{J}{\underset{j=1}{\sum }}\overset{K}{\underset{k=1}{\sum }}\overset{n_{ijk}}{\underset{t=1}{\sum }}\log\left(1-\pi_{ij}\right)r$$

$$\propto \;G(a_{0}-\;\overset{I}{\underset{i=1}{\sum }}\overset{J}{\underset{j=1}{\sum }}\overset{K}{\underset{k=1}{\sum }}\overset{n_{ijk}}{\underset{t=1}{\sum }}\log\left(1-\pi_{ij}\right),\;\frac{1}{b_{0}+\sum_{i=1}^{I}\sum_{j=1}^{J}\sum_{k=1}^{K}\sum_{t=1}^{n_{ijk}}L_{ijt}})\;\;$$ (A5)

According to Zhou *et al.* (2012), the conditional posterior distribution of $L_{ijkt}$ is a Chinese restaurant table (CRT) count random variable. That is, $L_{ijkt}∼CRT\left(y_{ijkt},r\right)\;$ and we can sample it as $L_{ijkt}=\Sigma_{l=1}^{y_{ijkt}}d_{l},$ where $\;d_{l}∼Bernoulli\left(\frac{r}{l-1+r}\right)\text{.}\;\;\;$ For details of the CRT random variable derivation, see Zhou and Carin (2012, 2015).

## Appendix B

### The Pólya-Gamma distribution

According to Polson *et al.* (2013), random variable ω has a Pólya-Gamma distribution with parameters b>0 and d∈R, denoted ω∼PG(b,d) if

$$\omega \overset{D}{=}\frac{1}{2\pi^{2}}\overset{\infty }{\underset{k=1}{\sum }}\frac{g_{k}}{{\left(k-\frac{1}{2}\right)}^{2}+d^{2}/(4\pi^{2})}$$ (B1)

where $g_{k}$ ∼ Gamma(b, 1) are independent gamma random variables, and D = indicates equality in distribution (Polson *et al.* 2013). However, it is not easy to simulate Pólya-Gamma random variables from Equation (B1), which is a sum of gamma random variables. To avoid the difficulties that can result from truncating the infinite sum given in Equation (B1), the density of a Pólya-Gamma random variable is expressed as an alternating-sign sum of inverse-Gaussian densities as:

$$P\left(\omega ;b,d\right)=\left\{\cosh^{b}\left(\frac{d}{2}\right)\right\}\;\;\frac{2^{b-1}}{\Gamma (b)}\overset{\infty }{\underset{k=0}{\sum }}{\left(-1\right)}^{n}\frac{\Gamma (n+b)(2n+b)}{\Gamma (n+1)\sqrt{2\pi \omega^{3}}}\exp(-\frac{{\left(2n+b\right)}^{2}}{8\omega }-\frac{d^{2}}{2}\omega$$ (B2)

where cosh denotes the hyperbolic cosine. A further useful fact is that all finite moments of a Pólya-Gamma random variable are available in closed form (Polson *et al.* 2013). In particular, the expectation may be calculated directly, and is equal to $E\left(\omega \right)=\frac{b}{2d}\tanh\left(\frac{d}{2}\right)$, where tanh denotes the hyperbolic tangent. Also, the Pólya-Gamma distribution is closed under convolution for random variates with the same scale parameter, given that if ${\text{ω}}_{1}∼\text{PG(}b_{1},\text{d)}$, and if ${\text{ω}}_{2}∼\text{PG(}b_{2},\text{d)}$ are independent, then $\omega_{1}+\omega_{2}∼\text{P G(}b_{1}+b_{2},\text{d)}$ (Polson *et al.* 2013). This is used to construct the proposed Gibbs sampler. More details on simulating Pólya-Gamma random variates can be found in section 4 of the paper of Polson *et al.* (2013). Also, it is important to point out that this method for simulating Pólya-Gamma random variables is implemented in the R package Bayeslogit.
